# Seasonal variation in the balance and strength of cooperative and competitive behavior in patches of blue mussels

**DOI:** 10.1371/journal.pone.0293142

**Published:** 2023-10-19

**Authors:** Jacob J. Capelle, Eva Hartog, Tony Wilkes, Tjeerd J. Bouma

**Affiliations:** 1 Wageningen University & Research -Wageningen Marine Research, Yerseke, The Netherlands; 2 HZ University of Applied Sciences, Vlissingen, The Netherlands; 3 Netherlands Institute for Sea Research, Yerseke, The Netherlands; 4 Faculty of Geosciences, Department of Physical Geography, Utrecht University, Utrecht, The Netherlands; King Abdulaziz University, SAUDI ARABIA

## Abstract

Aggregation into groups may affect performance of individuals through the balance and strength of facilitative versus competitive interactions. We studied *in situ* how seasonal variation in abiotic environment affects this balance for blue mussels, a semi-sessile species. We hypothesize that seasonal variation in stresses and resources affects the strength of the interaction. We expected that, in benign conditions (here: high food availability, medium temperatures, low hydrodynamic stress), performance is dominated by growth and is better at low densities, while at adverse conditions (here: low food availability, low or high temperatures, high hydrodynamic stress), performance is dominated by survival and higher at high densities. Mussels were kept in shallow subtidal exclosures at 10 different densities for a one-month period. This exact procedure was repeated seven times at the same location within a one-year period. We measured development in mussel patch shape, performance, and environmental parameters. Environmental conditions for mussels were most benign in summer and most adverse in winter. Patches developed into less complex shapes at lower densities, but also after stronger hydrodynamic disturbances. Towards summer, mussels became more active, aggregation behavior increased, and interactions became more pronounced. Towards winter, mussels became less active: aggregation behavior and growth rates declined and at the lowest temperatures survival started to decrease with mussel density. Survival and growth (by proxy of mussel condition) were both density-dependent; however, contrary to our expectations we found positive interactions between density and survival at the most benign conditions in summer and negative interactions at the most adverse conditions in winter. In between the two seasons, the strength of the interactions increased towards summer and decreased towards winter following a bell-shaped pattern. This pattern might be explained by the environmental mediated aggregation behavior of the mussels. The obvious seasonal pattern in balance and strength of density-dependent interactions demonstrates that strength and direction of intra-specific interactions are both strongly affected by environmental context.

## Introduction

Animals group together, from pairs up to large-scale aggregations, to gain potential communal benefits [1, 2], such as safety in numbers [3, 4], locating and defending food sources [5], thermoregulation [6, 7], defending offspring [8], and increasing immunoregulation [9, 10]. Group living may also have disadvantages for individuals within groups, when resources are limited and competition for food is high [11]. Therefore, the question of whether it is advantageous for an animal to join or to live in a group is highly context-dependent and may change as a function of environmental condition [12]. For most organisms, neither the communal benefits nor the competition strength is constant over different life stages and seasons, while conditions that are experienced in one period can have carry-over effects for the organism into the following period [13]. In mobile species, environmental mediated behavior is a strong determinant for the dynamics of group formation [14, 15], and densities may vary rapidly over time [16, 17]. In contrast, sedentary or semi-sedentary species have a limited ability to change group size at best, which means it is vital to optimize the density for facilitation and minimize competition.

Numerous studies have been designed to investigate effects of environmental conditions on the balance between competition and cooperation, since this is recognized as an important contributor to ecological theory [18, 19]. The stress gradient hypothesis (SGH) predicts that the role of positive interactions will be greater at increasing levels of stress [20]. A number of studies have underlined the importance of this hypothesis, but also stressed the conditional effect of environment and species [21–24]. Surprising few studies have addressed temporal effects, even though various parameters that contribute to environmental variation, are strongly affected by season [25]. We address this topic using blue mussels (*Mytilus edulis*) in a shallow subtidal environment as the model system.

Blue mussels are ideal model organisms in that they are semi-sedentary species that typically live and are adapted to dynamic and challenging environments. That is, the local environment may shift between benign and adverse, depending on the prevalent conditions that are often subjected to strong seasonal patterns. For mussels, benign can be defined as a situation with high food availability [26], medium temperatures [27], low turbidity [28], and low current and wave exposure [29], while adverse can be defined as the opposite to benign: low food availability, low or high temperatures, higher turbidity, and a higher exposure to current- and wave dynamics. Mussels show strong aggregation behavior, causing them to self-organize into spatial patterns. The dense patches that they form offer protection against dislodgement [30]. However, the formation of dense mussel patches can induce strong intra-specific competition [31–33]. Even in very small patches e.g., 21–28 individuals per patch it was already observed that mussels that were located in the center had a reduced growth and reproductive output, compared to mussels located at the edges or in isolated groups [33]. The extent to which patches are being formed depends strongly on the overall mussel density [30, 34]. By looking at mussel patch dynamics, we study how seasonal variation in the abiotic environment affects the balance between cooperation and competition, and how this depends on the overall mussel density.

**Fig 1:**
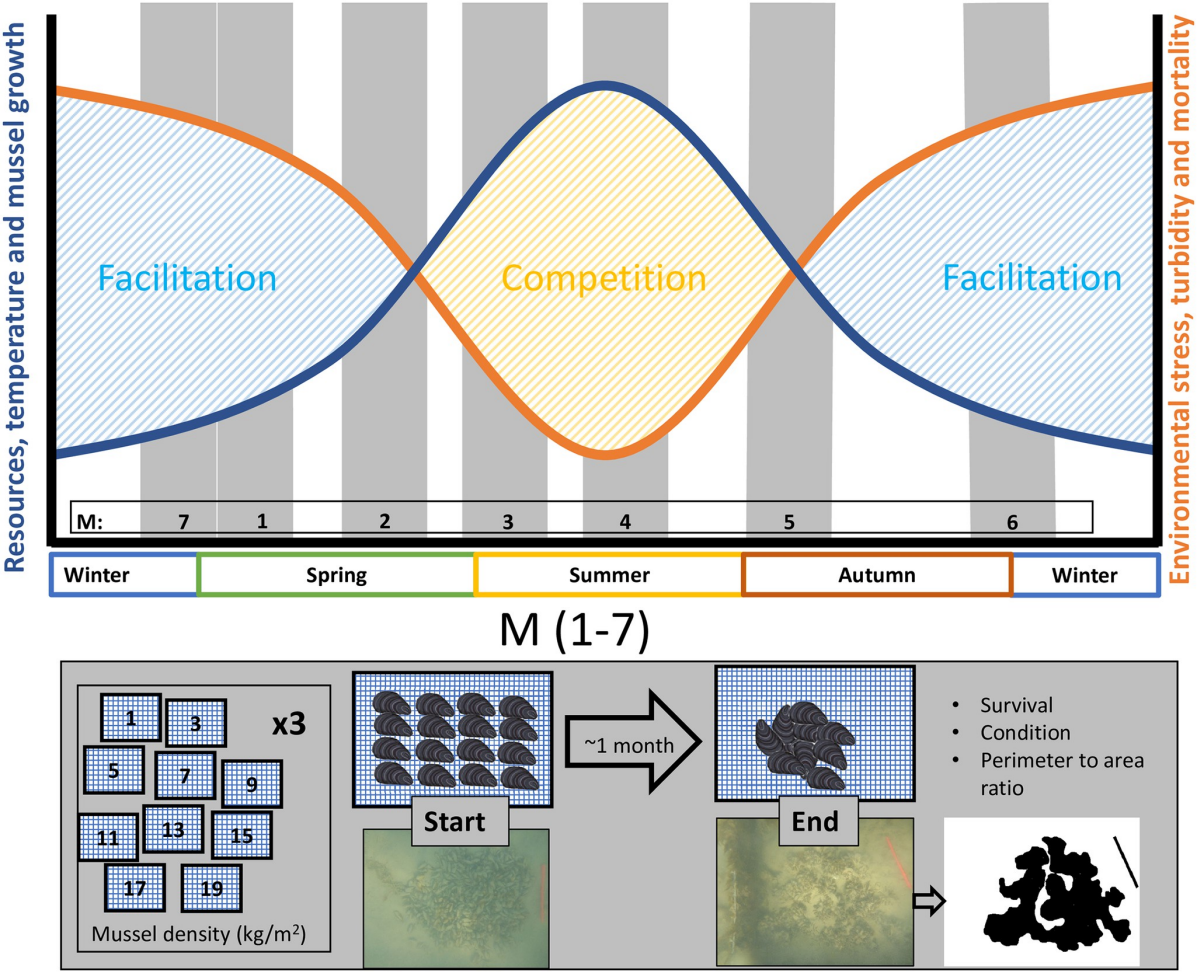
Conceptual drawing of hypotheses and methodology. **A**. Hypothetical interactions in subtidal mussel patches; in summer interactions are expected to be dominated by growth due to high level of resources and temperature (benign conditions). In winter interactions are expected to be dominated by survival or mortality, because of the high level of environmental stress, low resources and high turbidity (adverse conditions). Note that temperature is not considered a stressor under the subtidal conditions at the experimental area. We anticipate that in the balance between competition and facilitation, competition will play a more significant role when growth is dominant, while facilitation will be more important when survival is dominant and **B**. Method to map density dependent interactions; to map density-dependent interactions, mussels will be placed in cages at 10 different densities (in triplicate) from low to high and performance was monitored over approximately one month, at seven different time periods within a year (M1-7, indicated by the grey bars in A.). Photos illustrate this development (at the highest density of 19 kg/m^2^) and the binary image shows the mussel cover used to calculate the perimeter-to-area ratio.

Aggregation behavior in mussels is density-dependent; sparse clumping occurs at low densities and dense clumping at high densities [35, 36]. Therefore, we expect that competition (for resources), and cooperation (resilience against threats) increases with clump size. We hypothesize that seasonal variation in stresses and resources affect the strength between competition and cooperation: in more benign conditions, performance will be dominated by growth and mussels are expected to perform better at low densities, while in more adverse conditions, performance will be dominated by survival and is expected to be higher at high densities. The present study takes an experimental approach by studying how seasonal variation in the abiotic environment affect the balance and strength between cooperation and competition in a (semi-)sessile species. The main abiotic parameters that affect mussel performance are chlorophyll-a (a proxy for food), turbidity (a proxy for food quality and a proxy for hydrodynamic stress [37]), and temperature. Mussel performance was measured in the absence of predation over a period of about one month for a range of mussel densities and this procedure was repeated several times throughout the year. Positive density interactions–or cooperation–was indicated when either survival or condition increased with mussel density, while negative density interactions–or competition–was indicated when either survival or condition decreased with mussel density. Fig 1 illustrates a conceptual diagram depicting the hypotheses and the methods employed to test them.

## Material and methods

Mussels (*Mytilus edulis*) were kept in 10 different densities in predation-exclosures in the field for a period of about one month. This procedure was repeated seven times (runs) over a one-year period to cover all seasons (see Table 1). Relevant environmental parameters at the experimental site (that is, chlorophyl-a, turbidity, and temperature) were measured continuously for the duration of the experiments. Mussel growth, survival, and patch shape were calculated for each run.

**Table 1:** Mussel performance was evaluated in an experimental procedure that was repeated seven times over a one-year period. This table shows when the experiments took place and summarizes the conditions, by displaying the average and standard deviation over the respective period (W_0_ = average mussel weight at start, Temp = temperature, Chl-a = chlorophyll-a, Turb = turbidity).

Run	Start date	Duration, days	W_0_, g	Av. Temp, °C (95% CI)	Av. Chl-*a*, μg l^-1^ (95% CI)	Av. Turb., FTU (95% CI)
1	10/Mar/17	30	1.33	8.89 (0.55)	1.73 (0.09)	3.57 (0.72)
2	18/Apr/17	26	3.35	11.63 (0.31)	2.96 (0.55)	1.63 (0.32)
3	1/Jun/17	28	3.2	19.08 (0.44)	1.79 (0.14)	2.57 (0.70)
4	27/Jul/17	32	3.46	19.56 (0.10)	2.46 (0.12)	3.43 (0.20)
5	20/Sep/17	29	3.75	15.51 (0.26)	1.70 (0.11)	1.97 (0.17)
6	9/Nov/17	32	4.95	8.59 (0.59)	1.08 (0.05)	3.64 (0.87)
7	21/Feb/18	37	1.51	2.30 (0.46)	2.15 (0.46)	7.64 (2.61)

### Experimental set up, mussel response, and environmental variables

The experiment was conducted at a shallow subtidal soft sediment area in the Oosterschelde estuary, The Netherlands (51°33’29.8"N 3°54’02.1"E), at a tidal amplitude of about 3.5 m. The area is in use as mussel lease site and with permission of the owner of the plot no permits were required for the fieldwork. At low tide, water height above the sediment was 0.5 m on average. The set-up consisted of 30 randomly placed cages (0.55 m x 0.60 m x 0.25 m) at least 2 meters apart, completely covered with a 25 mm mesh. The mesh was placed to exclude predators and enclose mussels. The bottom of the cage was buried approximately 10 cm into the sediment to avoid the mussels attaching to the cage, rather than living on a soft bottom. At the onset of each run, mussels were homogeneously placed in 10 different densities (1, 3, 5, 7, 9, 11, 13, 15, 17 & 19 kg m^-2^ in an average density of 578, 1730, 2904, 4049, 5223, 6375, 7531, 8683, 9849 and 10981 mussels m^-2^, or 52, 156, 261, 364, 470, 574, 678, 781, 886, 988 mussels plot^-1^). We used a 0.3 x 0.3 m rectangle in the middle of each cage, to demark the area where mussels were added to create a buffer zone around the edges to prevent the mussels from attaching to the mesh. Each density was replicated three times. Densities were based on the range typically observed in the field, including the extremes [38], and were randomly assigned to the cages. Different mussels were used for each consecutive run. All mussels were collected at nearby locations and originated from seeded mussel culture plots in the same bay as where the experiment took place. Mussels were cleansed and sorted (in a range between 2 and 4 cm) prior to placement in the field.

Average wet weight, shell length, ash free dry weight (AFDW), and number of mussels were obtained from a subsample of all the mussels at the start of the experiment, to translate the starting weight of the mussels to number of individuals. The duration of each run was set for 30 days, but varied slightly because of weather and tide conditions (see Table 1). In previous studies, we found that the first month after relay is a critical period for survival and spatial organization is generally defined within one month [36, 38]. Growth rates during periods of low growth may be challenging to measure over a one month period. Therefore, we use mussel condition as a proxy for growth. Additionally, our previous research has shown a strong correlation between mussel meat weight and growth rate (see Fig 2 in [38]).

**Fig 2:**
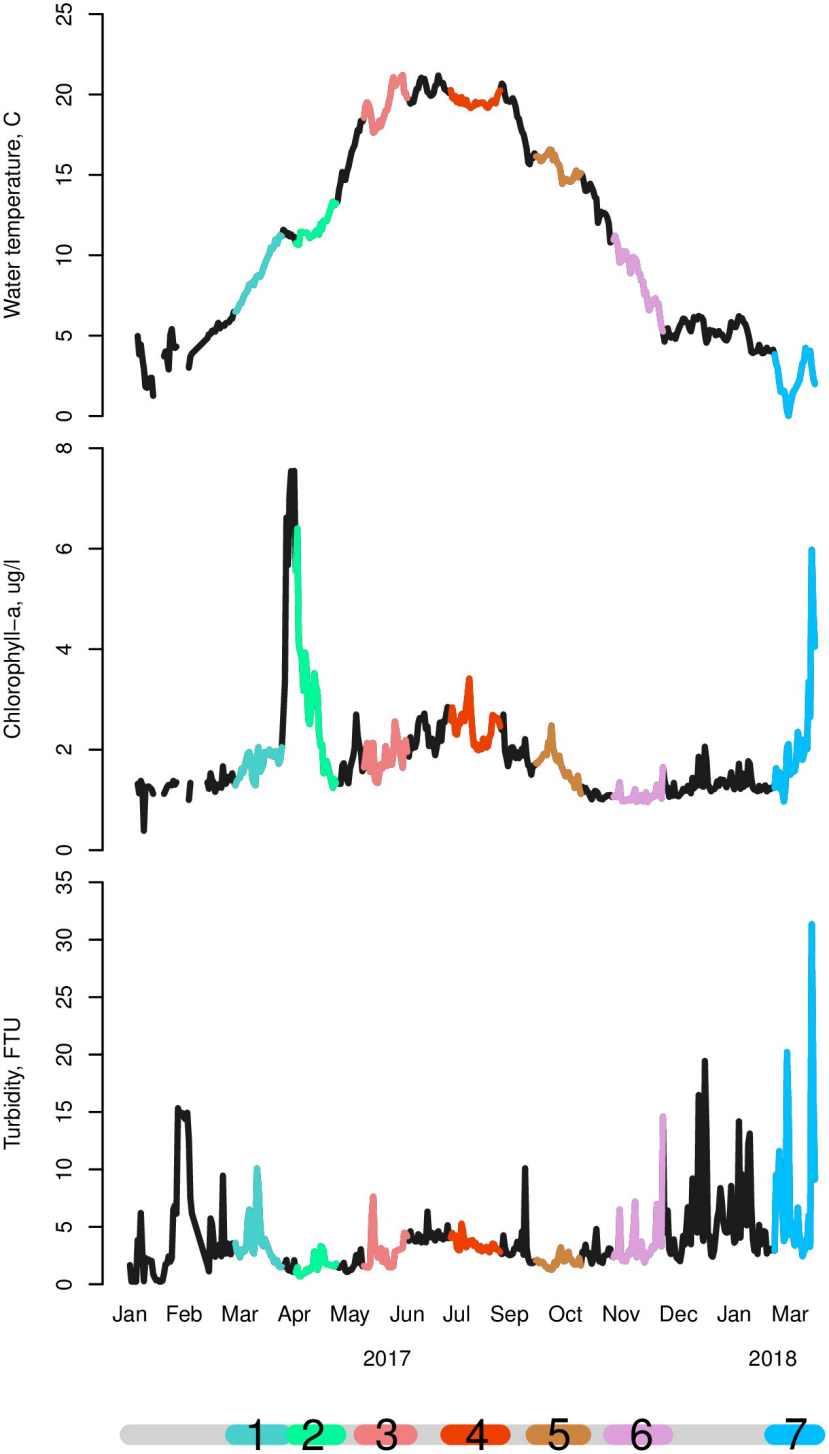
Environmental parameters measured at the experimental location, with daily averages calculated from 10-minute intervals. **A**. Water temperature (°C), **B**. Chlorophyll A (μg/l) and **C**. turbidity (FTU). Colors indicate the environmental parameters during the consecutive experimental runs, with in 2017: 1 = Mar 10^th^–Apr 9^th^, 2 = Apr 18^th^–May 15^th^, 3 = Jun 1^st^-27^th^, 4 = Jul 27^th^–Aug 28^th^, 5 = Sep 20^th^–Oct 19^th^, 6 = Nov 9^th^–Dec 11^th^ and in 2018: 7 = Feb 21^st^–Mar 19^th^.

At the end of an experimental run a top view photograph was taken from each cage. Mussels were collected and cleansed and total mussel mass was measured. Similar to the start of the experiment, from a homogenized subsample per cage wet weight, shell length, AFDW, and number of mussels were obtained.

A calibrated logger-type chlorophyll and turbidity meter (ACLW2-USB, JFE Advantech) measured chlorophyll-a (μg l^-1^), turbidity (FTU) and temperature (°C) in a 10-minute interval at the site during the entire experimental period (Fig 2). Chlorophyll-a is a reliable proxy for food quantity in the Oosterschelde [39]. In the analysis we did not consider effects of food quality.

### Calculations

Mussel density at the start (D_0_) and at the end (D_1_) was calculated by extrapolating the density:weight relations from subsamples to the total weight per cage. The condition index (CI) was calculated with the AFDW (mg) and the shell length (cm) as:

$$CI=\frac{AFDW}{L^{3}}$$ (1)

Condition index is used as a proxy for growth, because it is a more instantaneous measure than growth in weight or shell length over such a short time period. Top view photographs were analyzed in ImageJ [40]. Mussel-covered area was selected by hand, and mussels physically connected to each other were included in the same patch. The selection was saved as a binary image (see example in Fig 1). We placed a red piece of iron (15.3 cm) alongside the mussels when taking the picture as a size reference. Mussel covered area (mm^2^) and circumference (mm) were calculated from each picture and the perimeter-to-area ratio (PtoA) was defined as:

$$PtoA=\;\frac{Patch\;circumference}{Patch\;area}$$ (2)

Proportion of mussels that survived per cage was calculated for each run as:

$$p_{survival}=\frac{N_{1}}{N_{0}}$$ (3)

where N_0_ and N_1_ are the number of mussels at the beginning and the end of an experiment, respectively. There were some occasions where N_1_ was slightly greater than N_0_. Since there was no mussel recruitment, this is considered an estimation error and the analysis whenever N_1_ > N_0_, N_1_ was replaced with the number from N_0_.

### Statistical analysis

We corrected for differences in experimental duration assuming that the daily death rate within an experiment is constant throughout the experiment. The daily death rate was calculated by dividing the total number of deaths during an experiment by the duration of the experiment:

$$DailyDeathRate=\frac{N_{0}-N_{1}}{days}$$ (4)

The number of mussels that survived the experiment, out of the number of mussels that the experiment started with, was modeled with a quasi-binomial Generalized Linear Model (GLM) [41] using a logit link function (**Model 1**). With the assumption of a constant death rate, binomial failures, successes, and probabilities (*p*) are defined as follows:

$$binomial\;failures=\frac{N_{0}-N_{1}}{days}\;\times 22$$ (5)

$$binomial\;succesess=N_{0}-binomial\;failures$$ (6)

$$p=\frac{binomial\;successes}{N_{0}}$$ (7)

Using the assumption of a constant death rate, the daily death rate was multiplied by 22 (days) for mathematical consistency. New binomial successes and failures could become non-integer counts, but a quasi-binomial GLM can handle non-integer counts. The perimeter-to-area ratio (**Model 2**) and the Condition Index (**Model 3**) were both ratios and were modeled with linear models with log-transformed responses (see S1 File for details).

**Models 2** used the main effects: D_0_, mean turbidity, mean chlorophyll*-a*, and mean temperature. No interaction terms were present for these models, as that resulted in multicollinearity. The quasi-binomial model (**Model 1**) had these same main effects, as well as pair-wise interactions between mean turbidity, mean chlorophyll*-a* and mean temperature. **Model 3** (CI) used the main effects D_0_, mean turbidity, mean chlorophyll-a and temperature direction. Here, temperature direction is a categorical variable indicating how the temperature changed during the experiment. It has the following levels: “0” = the temperature was both high and stable. This is also the reference level, there was no “low and stable” level, as that never occurred during this study. “+” = the temperature increased from low to high; “-” = the temperature decreased from high to low. Temperature direction was used instead of mean temperature, as a model with mean temperature resulted in considerable non-linearity (correlogram of the data that was used for this model is attached as S3 Fig). No interaction terms were present for the final models as that resulted in multicollinearity. Pearson residuals were used to assess the model diagnostics for all linear and log-linear models. For the quasi-binomial GLMs, deviance residuals were used. The log-linear ratio models (models for the perimeter-to-area ratio and the Condition Index) were expected to have some heteroskedasticity. Therefore, robust standard errors, calculated using the “sandwich” R package [42–44], were used on the log-linear ratio models to correct the effects that heteroskedasticity may have on the variances and thus *p*-values of the covariates. There were no clear violations of the model assumptions, and the fit of the models was reasonably good.

Holm’s correction for multiple comparisons [45] was applied to *p-*values of the coefficient significance tests to correct for multiple testing. Corrected *p*-values lower than 0.05 were considered significant. All statistical analysis were performed in R and RStudio [46, 47].

## Results

### Environmental characterization

Environmental patterns for Water temperature, chlorophyll-a, and turbidity over the period in which the consecutive experimental runs took place followed a typical annual trend for the region where the experiment was performed (Fig 2). Water temperature differences between summer and winter were about 20°C. Turbidity showed higher values from November to March, which is consistent with more storms causing upstirring of sediment in the fall-winter season. Chlorophyll-a increases sharply in spring due to the spring bloom that occurs in the North Sea-dominated region [48], then remains elevated over summer and decreases over autumn to reach lowest levels in winter. Because the mussels were continuously submerged no extreme temperatures were recorded, impact of heatwaves in the same region is limited to tidal flats [49].

### Mussel survival

**Fig 3:**
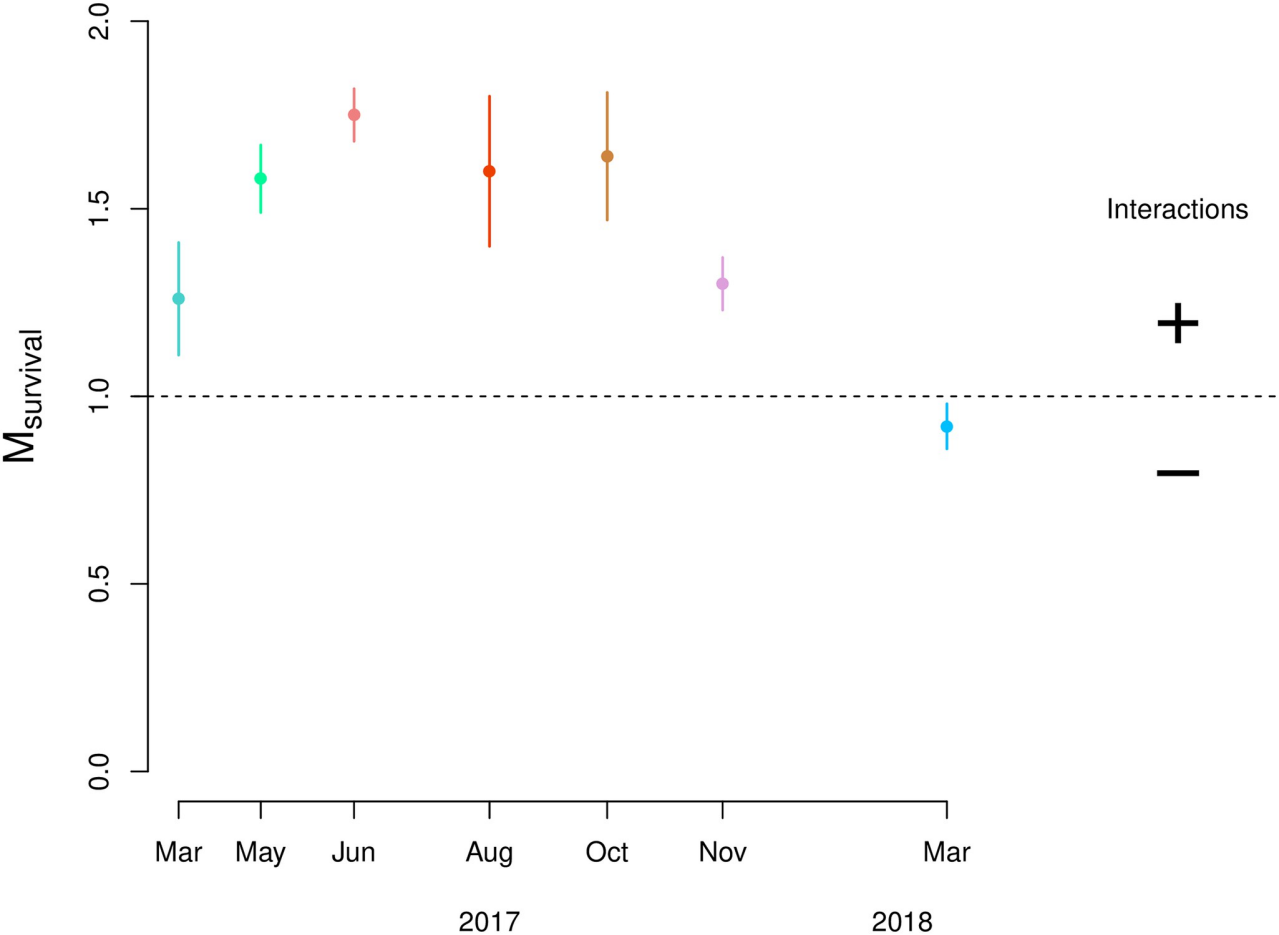
Slope coefficient (M_survival_± SE, n = 30, a proxy for the strength of a density-dependent effect) of the log-log relation between end density and starting density for each consecutive experimental run: ln[D_1_] = M_survival_ • ln[N_0_]+b. Each run consisted of 10 different densities in triplicate. When the slope is below 1, a negative interaction between the individuals is expected (indication of competition), because the chance of survival decreases with mussel density. When the slope is above 1, positive interactions are expected (indication of cooperation) because the chance of survival increases with mussel density.

In line with our hypothesis, we found that overall an increase in density corresponds to an increase in the odds of survival if all other covariates remain the same (Table 2). The strength of the density effect was quantified using the slope (M_survival_) of the log-log relation between end-density (*D*_*end*_) and starting-density (*D*_*0*_), with a slope below 1 indicating a negative density effect and a slope above 1 indicating a positive density effect. The slope for sequential experimental runs throughout the season shows a bell-shaped pattern (Fig 3) indicating the presence of a strong seasonal effect. Underlying plots are attached as supplementary material (S1 Fig).

**Table 2:** Summary results of Model 1: Survival of mussels. Dispersion parameter is 17.37, D_0_ = starting density, Temp = temperature (°C), Chl-*a* = chlorophyll *a*, Turb = turbidity, used as proxy for hydrodynamics stress, n.s. = non-significant.

*Mussel survival*			
Term	Estimate	Std. error	p.value
(Intercept)	15.44	1.52	<0.001
D_0_	0.034	0.007	<0.001
Temp.	-0.68	0.061	<0.001
Chl-*a*	-9.12	0.69	<0.001
Turb.	-0.10	0.42	0.80 (n.s.)
Temp:Chl-*a*	0.58	0.044	<0.001
Temp:Turb	-0.16	0.013	<0.001
Chl-*a*:Turb	0.42	0.16	0.02

**Fig 4:**
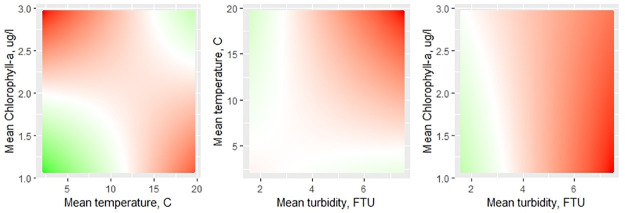
Marginal and interaction effects of the temperature, chlorophyll-a, and turbidity on the odds of survival. The color indicates the relative effect of the interaction terms on the linear predictions: more green means a higher survival chance (higher log-odds), more red means a lower survival chance (lower log-odds). More white means the relative effect is closer to 0 (neither increase nor decrease in log-odds).

Contrary to our expectations, survival chances were lower at higher temperatures and at higher chlorophyll-a values (Table 2). Also, the interaction effects between temperature with respectively chlorophyll-a and turbidity and between chlorophyll-a and turbidity were significant (Table 2). Fig 4 shows both the marginal and the interaction effects of the temperature, chlorophyll-a, and turbidity on survival chances. Chance of survival is higher when extremes in temperature and chlorophyll-a levels concur; that is, at the combination of low temperatures and low chlorophyll-a levels and at the combination of high temperatures and high chlorophyll-a levels. The latter combination refers to monitoring moment 4 (Fig 2) and although a strong density effect is observed at that moment (Fig 3) overall mortality was lower. Survival chance was lower at higher turbidity levels, which is a proxy for hydrodynamic stress, especially at higher temperatures and lower chlorophyll-a levels.

### Perimeter-to-area ratio

**Fig 5:**
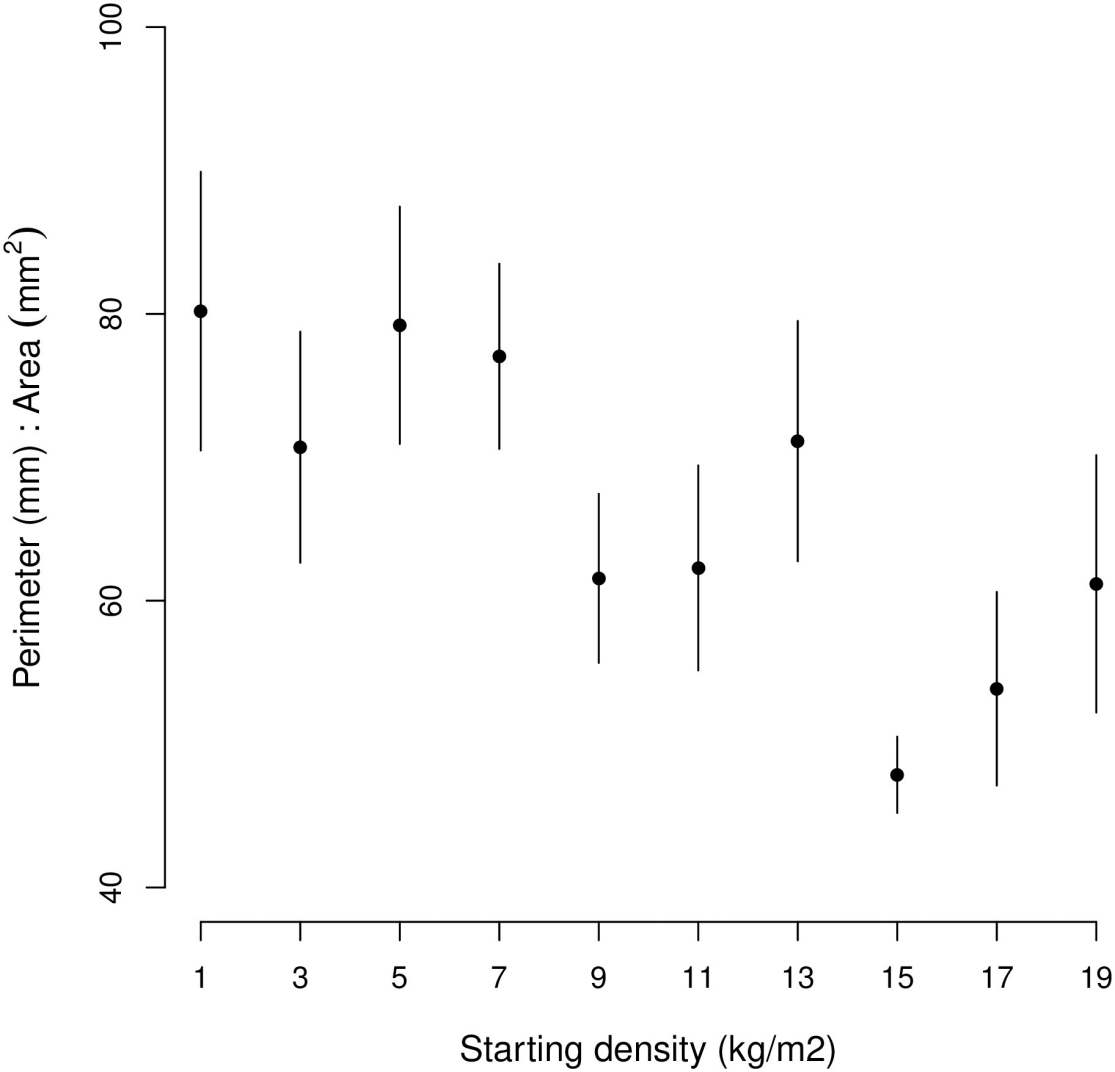
Perimeter: Area ratio ± SE (n = 21) of the mussel patches at the end of the experimental run as a function starting density.

An increase in starting density corresponds to a decrease in perimeter-to-area ratio, which confirms our hypothesis (Table 3). This indicates that, at higher starting densities, mussels aggregated quickly into larger and more complex patches (Fig 5). Of all the environmental parameters, only turbidity had a positive effect on this ratio: a higher turbidity resulted in an increase in the perimeter-to-area ratio, indicating that mussels aggregated less into larger complex patches. Since turbidity is a proxy for hydrodynamic stress, our findings indicate that an increase in hydrodynamic stress, as occurs during storms driving turbidity, results in the breaking up of mussel patches.

**Table 3:** Summary results of Model 2: Perimeter-to-Area ratio, D_0_ = starting density, Temp = temperature (°C), Chl-*a* = chlorophyll *a*, Turb = turbidity, used as proxy for hydrodynamics stress, n.s = non-significant.

*Perimeter-to-Area ratio*			
*Term*	*Estimate*	*Std*. *error*	*p*.*value*
*(Intercept)*	*3*.*83*	*0*.*22*	<0.001
D_0_	*-0*.*02*	*0*.*007*	*0*.*007*
Temp.	*0*.*01*	*0*.*01*	*0*.*43 (n*.*s*.*)*
Chl-*a*	*-0*.*24*	*0*.*12*	*0*.*10 (n*.*s)*
Turb.	*0*.*19*	*0*.*05*	*0*.*002*

### Mussel condition

**Fig 6:**
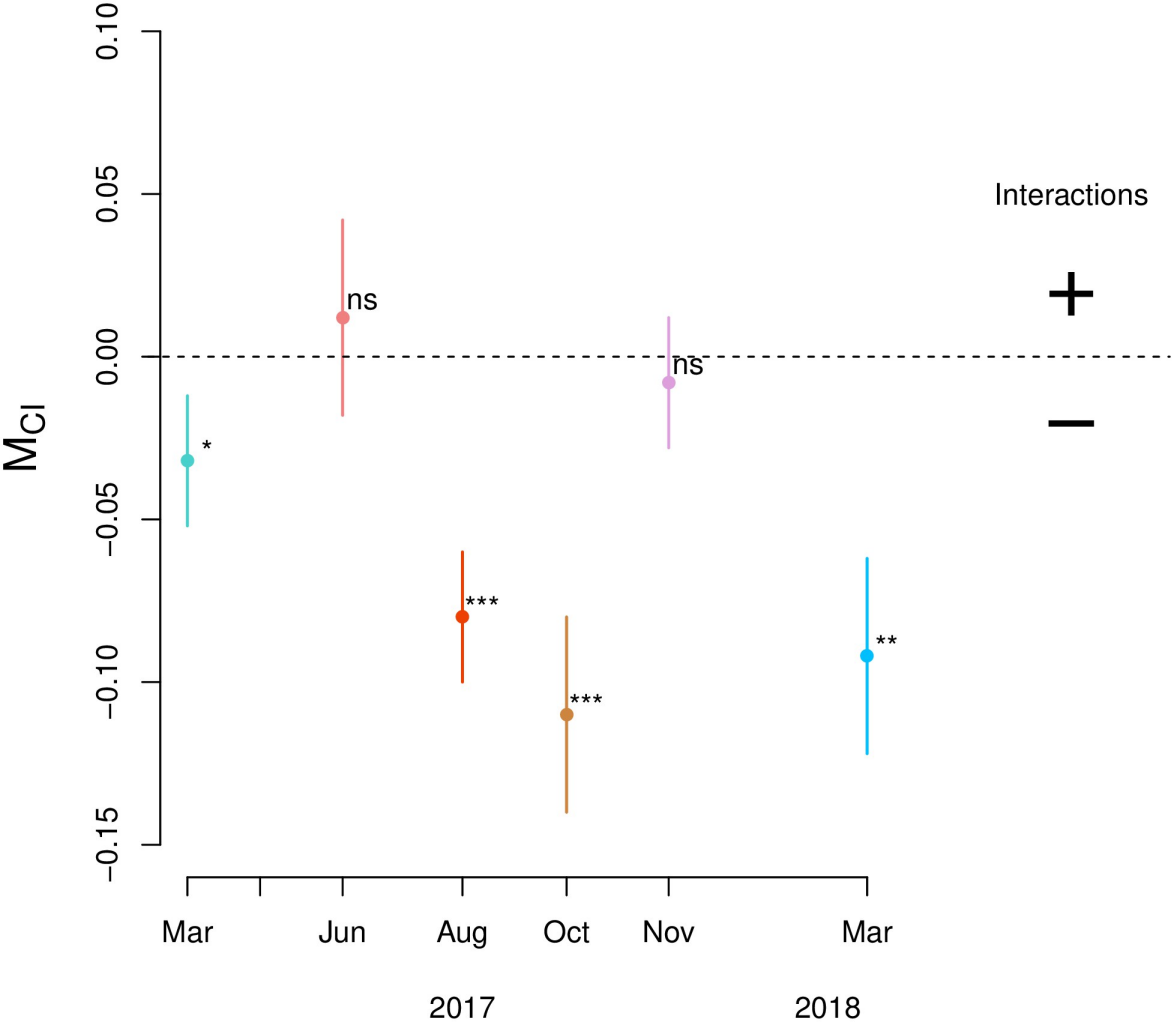
Slope coefficient M_CI_ ± SE (n = 30) between the log-log relation between mussel condition index (mg/cm^3^) and starting biomass for the different consecutive runs, as ln[CI] = M_CI_ • ln[D_0_]+b., each run consisted of 10 different densities (ns = not significant, *p<0.05, **p<0.01, ***p<0.001). Run 2 is not included due to a lack of data. At negative M_CI_ values mussel conditions decreases with mussel density (negative interactions) indicating competition for food, at positive M_CI_ values mussel condition increases with mussel density (positive interactions—not observed).

An increase in density corresponds to a decrease in the condition, as represented by the condition index (CI; Table 4). A stable and high temperature during the experiment (TempDirection = 0) corresponds to a higher condition index than an increasing or decreasing temperature. Experiments where the temperature decreased from high to low had a higher condition index than experiments where the temperature increased from low to high. Temperature direction is more an indication of season than it is of actual temperature (see also Fig 2). Turbidity also had a significant negative effect on the mussel condition (Table 4). The slope between CI and starting density indicates, for all significant cases, a negative density dependence of the condition index (S2 Fig, Fig 6). This relation means that mussels are less fit when growing at higher densities and is valid across the various periods of the year.

**Table 4:** Summary results of Model 3: Condition index (mg/cm^3^), D_0_ = starting density, Temp Dir = temperature (°C) direction (- = decreasing, 0 = stable, + = increasing), Chl-a = chlorophyll a, Turb = turbidity, used as proxy for hydrodynamics stress, n.s = non-significant.

*Mussel condition*			
*Term*	*Estimate*	*Std*. *error*	*p*.*value*
*(Intercept)*	*1*.*82*	*0*.*12*	<0.001
D_0_	*-0*.*007*	*0*.*002*	<0.001
Temp Dir -	*-0*.*30*	*0*.*11*	*0*.*01*
Temp Dir +	*-0*.*33*	*0*.*05*	<0.001
Chl-*a*	*0*.*12*	*0*.*08*	*0*.*13 (n*.*s*.*)*
Turb.	*-0*.*09*	*0*.*02*	<0.001

## Discussion

The density-dependent patterns in mussel growth, mussel survival, and mussel aggregation were affected by environmental context in that the strength of the density effect followed a seasonal pattern that mimics the environmental trend in temperature and chlorophyll-a (a proxy for the available food quantity). We expected that, at conditions considered benign for mussels, such as when food availability is high and at medium temperatures, a negative density effect would occur; that is, a high growth rate and some degree of competition. Surprisingly, although growth (mussel condition) was indeed higher at more benign conditions, survival increased with mussel density. At more adverse conditions for mussels that typically occur more frequent in winter, such as low food conditions, low temperatures, and a higher frequency of hydrodynamic stress, we observed at the lowest temperature that mussel survival decreased at higher densities.

### Balance between competition and cooperation

Species will experience concurrent cooperation and competition throughout their development [50] and at different environmental conditions [51]. There is a body of empirical evidence that, in intra-specific interactions, the balance between cooperation and competition shifts from positive (cooperation) to negative (competition) when the environment shifts from benign to harsh (e.g., [19–21, 23, 52, 53]. These findings seem to oppose the basics of the original stress gradient hypothesis (SGH), which predicts that cooperation will dominate under stressful environments and competition will dominate under benign environments [18, 20]. Ever since this theory was harnessed in the SGH, discussions have arisen regarding the conditional validity of this theory, particularly about the role of cooperation and several studies refined the SGH with the addition that cooperation is underestimated under benign conditions [22, 54–56]. For instance, organisms are more sensitive to stress under more benign conditions, while at high stress levels species tend to be more dependent on their feedback mechanisms. Our study deals with typical semi-sessile ecosystem engineering species. Ecosystem engineers have evolved to ameliorate stress under harsh conditions and alleviate limiting abiotic and biotic stresses for a range of species [57]. At adverse conditions, feedback from the engineer, such as aggregation into patterns [58], promotes survival, although it also lowers production. When the environment becomes more benign, activity increases and, in our study, resulted in a behavior that increases mortality, due to density-dependent competition for food and density-dependent cooperation for survival, all at the same time.

### Effects of intra-specific interactions on survival

Which factors might be responsible for this observed behavior? In order to test only for the effect of the abiotic environmental parameters, predation was excluded. The main abiotic environmental parameters that do affect bivalve population dynamics in the subtidal zone are change in temperature [59], food availability [60], sediment dynamics [61], and hydrodynamics [62]. All of those parameters were measured directly or by proxy. Apparently, competition for food reduced mussel condition, but it did not accelerate mussel mortality. In soft sediment habitats, mussels need each other to prevent burial, a risk that increases with mussel cover [63]. Observations during retrieving the mussels from the experiment suggested that, in the low densities, where the mussels were organized in very small patches, mussels were often covered with sediment. At higher densities, mussels crawled on top of each other or aggregated in an interconnected pattern that is elevated from/laying on top of the sediment (see also Fig 5). This hypothesis is strengthened by the finding that *M*. *edulis* mortality caused by burial increases with temperature [64], probably due to higher oxygen demands at higher temperatures and is therefore expected to be higher in summer.

### Effect of environmental context on the cooperation and competition

Mussel condition declined with mussel density, and, at the same time, survival interacted positively with density, meaning that competition concurred with cooperation. Density also affected the spatial structure of the mussel patches; at lower densities, patches were more complex (as in a higher perimeter:area, see also [65]). Furthermore, higher turbidity related to more complex patches, an effect that is expected when patches are broken up by hydrodynamic disturbances, such as during storms.

Environmental patterns followed a normal annual trend for the region in which the experiment was performed. The pronounced effects of temperature on the balance between cooperation and competition are interesting parameters to consider in scenario studies on climate change. In our experiment, the temperature did not reach levels that can be considered stressful for the mussels; instead, it increased cooperation between mussels. At higher temperatures, intra-specific interactions might buffer mortality at higher densities. But what will happen when temperature becomes a stressor? Will the balance between competition and cooperation shift towards more competitiveness, as observed in our study where conditions become more adverse, in that case Fig 2 may become bimodal. For instance, in a recent study on Baltic mussels, density dependency was presented as an accelerator of reduced population growth under increasing temperature scenarios [66].

It would also be interesting to include inter-specific effects. For example, the two most common predators for subtidal mussels in the study area are starfish and shore crabs, both of which show temperature-dependent feeding rates [67, 68] and interact with the (density-mediated) level of aggregation of the prey [69, 70]. That leads to the hypothesis that the effects we found in our study might even become more pronounced when inter-specific effects are included.

Sessile ecosystem engineers are unable to move to benign environments and depend on their feedback to optimize environmental dependent growth and survival. The coastal environment is highly dynamic over time and the species have adapted to short time variation in the environment, such as tidal effects [71], but also to longer term variation, such as in seasonal changes [72]. When conditions are harsh for the species (such as in winter, when temperatures are low, food is scarce, and disturbances by storms are frequent), ecosystem engineers show low levels of activity and increase survival through their feedback mechanisms [29]. When conditions get better in spring, activity will increase and will peak during summer when the environment is most benign (that is, when there are high temperatures, medium food levels, and low disturbance probability). Our results show that a high level of activity increases the competition for food, but also increases behavioral cooperation. In our case this is most likely done by actively using conspecifics to maneuver to a beneficial position within the patch.

An important implication of our study is that it is vital to take the environmental context into account when studying and discussing density-dependent patterns and performance. The strength of the cooperation-competition balance changes over the season. We repeated the same set-up seven times over a year and would jump to very different conclusions from isolated experiments when performed in summer (density-dependent facilitation in survival), than when performed in winter (density-dependent competition in survival).

## Supporting information

S1 File Modelling ratios where numerator and denominator are correlated. (DOCX)

S1 Fig Log-log relation between end density and starting density per run. (DOCX)

S2 Fig Log-log relation between Condition index and starting biomass per run. (DOCX)

S3 Fig Correlogram for data used in model 3. (DOCX)
