# Supplementary material for: Seasonal variation in the balance and strength of cooperative and competitive behavior in patches of blue mussels
Source: PLoS One. 2023 Oct 19;18(10):e0293142. doi: 10.1371/journal.pone.0293142 (PMC10586602; doi:10.1371/journal.pone.0293142)
Supplement: S1 File — (DOCX) [file pone.0293142.s001.docx]

***S1 Modelling ratios where numerator and denominator are correlated (for perimeter to area ratio and condition index)***

Having a ratio as a response variable in a model, where the numerator and denominator of the ratio are correlated (for example: in the ratio weight/length, the numerator and denominator are clearly correlated) makes the model quite difficult to work with. It is better to just remove the ratio altogether. This section explains how the ratio response variable in a linear model was transformed to remove the ratio. The methodology explained in this section was applied to the model for the perimeter-to-area ratio and the model for the condition index.

Suppose one has a linear model that can be described as follows:

$$\frac{Y}{Z^{a}}=X\beta+\varepsilon$$

As in standard linear model notation, X is the model matrix, β is the vector of coefficients, and *ε~Norm(0, σ_ε_)* is the error term.

One way to get rid of the explicit ratio is through the following two steps. The first step is to take the logarithm of the response, as this turns the division into a subtraction (which is easier):

$$\log\left( Y \right)-a\log\left( Z \right)=X\beta+\varepsilon$$

The second step is to bring a log(Z) to the other side, simply by adding *+a log(Z)* to both sides:

$$\log\left( Y \right)=a\log\left( Z \right)+X\beta+\varepsilon$$

Notice that a *log(Z)* has become the offset term. It is important to note that, in the following two equations,

$\log\left( Y \right)=a\log\left( Z \right)+ X\beta$*,* and $\log\left( \frac{Y}{Z^{a}} \right)=X\beta$*,* the vectors of coefficients, are the same (safe for some rounding errors), and have the same interpretation; in both models, Xβ is equal to the expected value of log(Y/Z^a^). The difference lies in the definition of the residuals. This is the essence of the solution: The simple interpretation of the values is kept, the predictions are computed ignoring the offset term, but the residuals used are based on the model with the offset term. We model a ratio, but with fewer diagnostic and variance problems that come with modeling a ratio.

Predictions for the untransformed response (so without the logarithm) are computed as:

$$E\left( \frac{Y}{Z^{a}} \right)=exp(X\beta)$$

Using the method described above, one can formulate the model for the perimeter-to-area ratio as follows:

$$\log\left( Perimeter \right)=X\beta+1\times\log\left( Area \right)+\varepsilon$$

The model for the condition index can be formulated as follows:

$$\log\left( AFDW \right)=X\beta+3\times\log\left( L \right)+\varepsilon$$
