# Supplementary material for: Seasonal variation in the balance and strength of cooperative and competitive behavior in patches of blue mussels
Source: PLoS One. 2023 Oct 19;18(10):e0293142. doi: 10.1371/journal.pone.0293142 (PMC10586602; doi:10.1371/journal.pone.0293142)
Supplement: S2 Fig — (DOCX) [file pone.0293142.s003.docx]

***S3 Figure. Log-log relation between Condition index and starting biomass per run with M_CI_=slope coefficient (n.s = not significant, *=p<0.05, **=p<0.01.***p<0.001)***

**
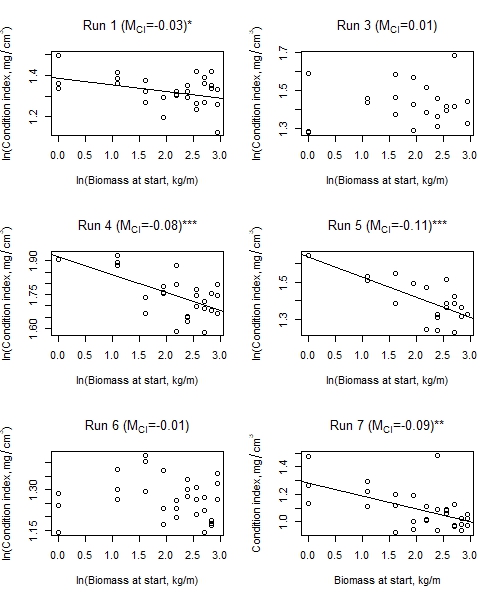
**
