# Supplementary material for: Seasonal variation in the balance and strength of cooperative and competitive behavior in patches of blue mussels
Source: PLoS One. 2023 Oct 19;18(10):e0293142. doi: 10.1371/journal.pone.0293142 (PMC10586602; doi:10.1371/journal.pone.0293142)

***S4 Figure. Correlogram for data used in model 3: CI = condition index (mg/cm^3^), meanChla = mean chlorophyll-a (µg/L), meanTurb = mean turbidity (FTU), meanTemp = mean temperature (⁰C),***

*GrowthRate = growth rate over experimental run (g/day), month.num = number of the month, Period_as.num = index of period, PtoA = Perimeter-to-area ratio (m^-1^), Days = duration of the run, Perimeter and Area = resp. total perimeter (m) and total area (m^2^) of all mussel patches at the end of the run, DensityStart = number of mussels at the start of the experiment (/m^2^), Treatment = index for the different densities and runs, L.cm = mussel length at the end of the run (mm), ADFW.mg = ash free dryweight of mussels at the end of the run (g).*


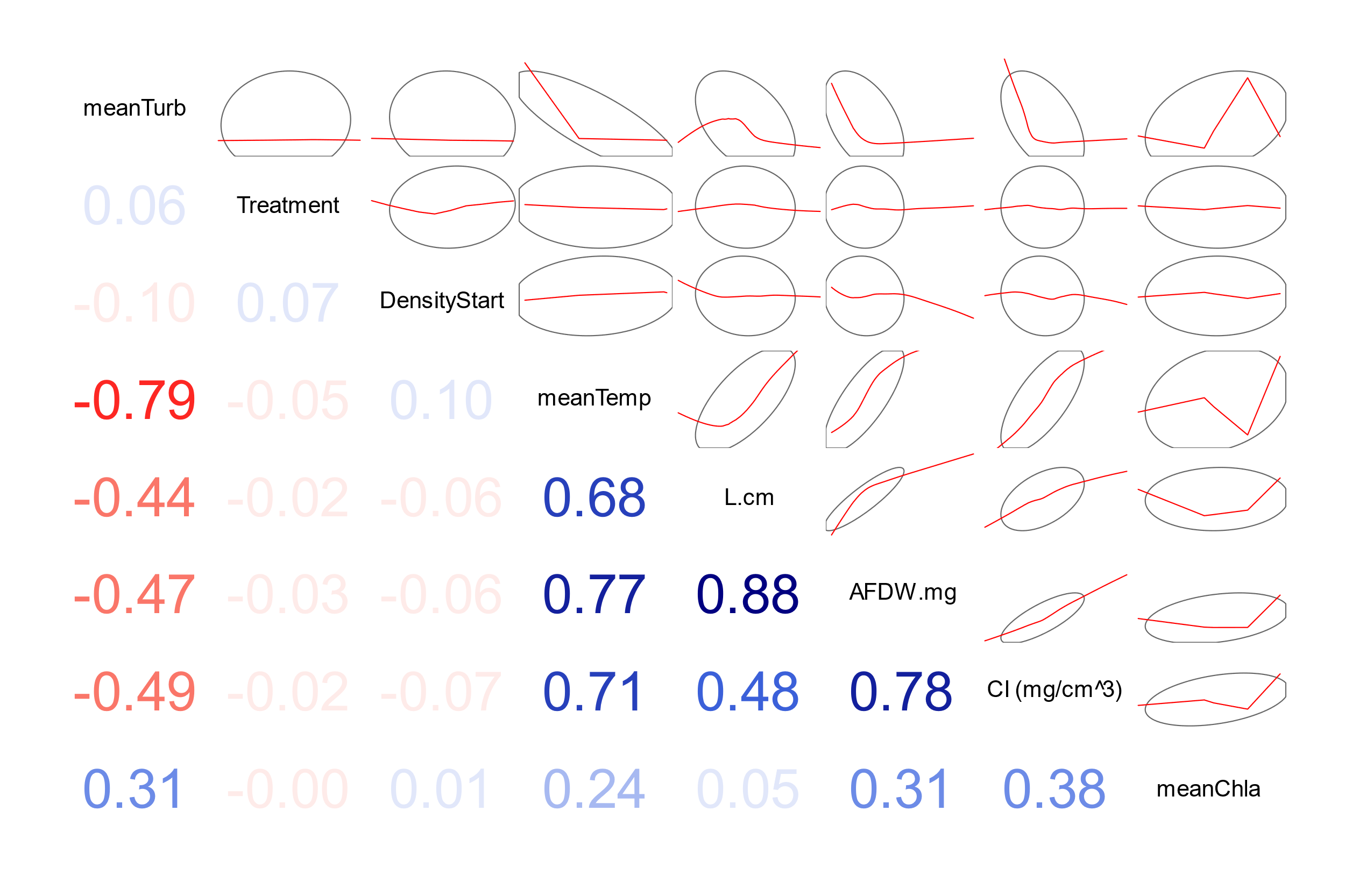

Supplement: S3 Fig — (DOCX) [file pone.0293142.s004.docx]
